# Effect of Protein O-Mannosyltransferase (MSMEG_5447) on *M. smegmatis* and Its Survival in Macrophages

**DOI:** 10.3389/fmicb.2021.657726

**Published:** 2021-06-30

**Authors:** Liqiu Jia, Shanshan Sha, Shufeng Yang, Ayaz Taj, Yufang Ma

**Affiliations:** ^1^Department of Biochemistry and Molecular Biology, College of Basic Medical Sciences, Dalian Medical University, Dalian, China; ^2^Department of Microbiology, College of Basic Medical Sciences, Dalian Medical University, Dalian, China

**Keywords:** host-pathogen interaction, O-mannosylation, protein O-mannosyltransferase, *Mycobacterium smegmatis*, phagosome–lysosome fusion

## Abstract

Protein O-mannosyltransferase (PMT) catalyzes an initial step of protein O-mannosylation of *Mycobacterium tuberculosis* (Mtb) and plays a crucial role for Mtb survival in the host. To better understand the role of PMT in the host innate immune response during mycobacterial infection, in this study, we utilized *Mycobacterium smegmatis* pmt (*MSMEG_5447*) gene knockout strain, ΔM5447, to infect THP-1 cells. Our results revealed that the lack of *MSMEG_5447* not only impaired the growth of *M. smegmatis* in 7H9 medium but also reduced the resistance of *M. smegmatis* against lysozyme and acidic stress *in vitro*. Macrophage infection assay showed that ΔM5447 displayed attenuated growth in macrophages at 24 h post-infection. The production of TNF-α and IL-6 and the activation of transcription factor NF-κB were decreased in ΔM5447-infected macrophages, which were further confirmed by transcriptomic analysis. Moreover, ΔM5447 failed to inhibit phagosome–lysosome fusion in macrophages. These findings revealed that PMT played a role in modulating the innate immune responses of the host, which broaden our understanding for functions of protein O-mannosylation in mycobacterium–host interaction.

## Introduction

*Tuberculosis* (TB), caused by *Mycobacterium tuberculosis* (Mtb), is one of the top 10 causes of death and the leading cause of death from a single infectious agent. Today, the burden of TB is still in a phase of high level, largely due to the TB/HIV coinfection and emergence of high drug-resistant Mtb strains (WHO, 2020). Under this situation, efforts for understanding pathogenicity of Mtb become eagerly important.

The unique cell envelope of Mtb is composed of peptidoglycan, arabinogalactan, mycolic acids, phosphatidylinositol mannosides, lipomannans, lipoarabinomannans, and proteins. It not only provides an impermeable barrier for antimicrobial drugs resistance but also is crucial for Mtb pathogenicity and survival in infected host (Abrahams and Besra, 2016; Turner and Torrelles, 2018). Recent studies showed that many cell envelope proteins and secreted proteins of Mtb were frequently O-mannosylated (Espitia et al., 2010; Mehaffy et al., 2019). For example, 41 putative O-mannosylated proteins in Mtb culture filtrate were identified *via* concanavalin A (ConA) lectin-specific two-dimensional gel electrophoresis (Gonzalez-Zamorano et al., 2009). Tucci et al. (2020) identified 46 O-glycoproteins from culture filtrate of Mtb by LC-MS/MS, and most of those proteins are involved in intermediary metabolism and respiration, as well as cell wall (CW) and cell process according to the Mtb database. The biological roles of Mtb glycoproteins also have been investigated currently. It has been reported that O-mannosylation of protein Mtb had closely linked with protein properties including the activity, subcellular localization, and stability of proteins and the permeability of the CW (Herrmann et al., 1996; Sartain and Belisle, 2009; Arya et al., 2013; Rolain et al., 2013). Additionally, O-mannosylation of protein displayed a profound impact on host–pathogen interaction, such as receptor recognition, immunomodulation, antigenicity, and Mtb pathogenicity (Loke et al., 2016; Vinod et al., 2020). For example, O-mannosylated proteins Apa, LpqH, and PstS1 as adhesins bound with c-type lectins to achieve cell adhesion, facilitating subsequent establishment of infection (Diaz-Silvestre et al., 2005; Ragas et al., 2007; Esparza et al., 2015). Further studies revealed that natural O-mannosylation of Apa is crucial for stimulating the T cell antigenicity and dendritic cell-mediated T cell polarization (Horn et al., 1999; Pitarque et al., 2005; Nandakumar et al., 2013). Recently, the protective capacity of *Mycobacterium bovis* BCG was also improved by boosting with the O-mannosylated protein of BCG (Deng et al., 2020).

Protein O-mannosyltransferase (PMT) catalyzes the initial step of protein mannosylation by transferring the mannosyl residue to serine or threonine residue of proteins. VanderVen et al. (2005) first identified Rv1002c as Mtb PMT because overexpression of Rv1002c in *Mycobacterium smegmatis* increased the PMT activity of membrane fractions *in vitro*. Increased interest in O-mannosylation stemmed from the fact that the absence of Rv1002c had greatly reduced the Mtb survival in mice (Liu et al., 2013). Recent studies showed that PMT related to the release of lipoarabinomannan (LAM) and affected host inflammatory responses (Alonso et al., 2017). Even though these findings endow PMT with potential as a drug-targetable virulence factor in host–pathogen interactions, its physiological role in Mtb and its biological role in innate immunity of the host are still poorly characterized.

**FIGURE 1:**
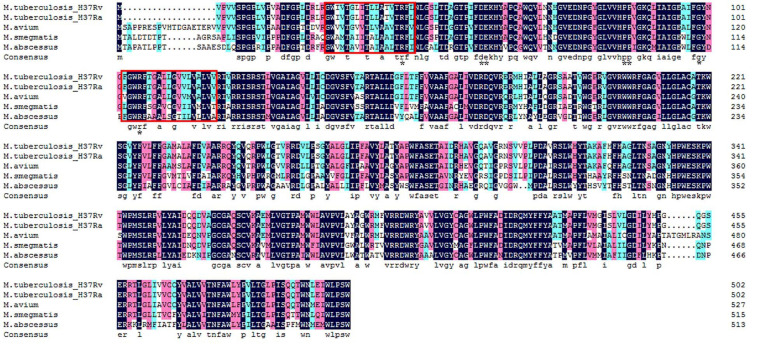
Amino acid multiple-sequence alignment of O-mannosyltransferase in mycobacterium species. GenBank accession numbers: *M. tuberculosis* H37Rv: CCP43752; *M. tuberculosis* H37Ra: ABQ72745; *Mycobacterium avium*: ANR90417; *M. smegmatis*: AFP41739; and *M. abscessus*: WP_005059031. The dark blue box indicates 100% identical of amino acid sequence among five mycobacterium strains. The pink and sky blue colors indicate >75 and >50% similarity of amino acid sequence among five mycobacterium strains, respectively. Two functional transmembrane domains of PMT were framed by the red box, and the conserved PMT active site residues were marked with the black star based on the VanderVen study in 2005. Alignment was conducted using DNAMAN software.

*Mycobacterium smegmatis* is a convenient model for the study of PMT due to its ability to produce glycosylated Mtb recombinant proteins (Bashiri and Baker, 2015). It is also an important tool as a vaccine vector in expressing heterologous proteins (Triccas and Ryan, 2009). Additionally, *MSMEG_5447*, a gene that encodes PMT in *M. smegmatis*, is homologous with Rv1002c and conserved among mycobacteria (Figure 1). In our previous work, an *M. smegmatis* mutant strain with *MSMEG_5447* gene disruption, ΔM5447, was constructed and confirmed by obtaining non-mannosylated protein Rv0431 which is a mannosylated protein in Mtb (Deng et al., 2016). In this study, MSMEG_5447 complementary strain was generated by transforming the pMind-MSMEG_5447 plasmid to the ΔM5447 strain. The impact of PMT on *M. smegmatis* viability under stress conditions was measured, and the invasion and survival of ΔM5447 in the human macrophage cell line THP-1 were evaluated. The level of inflammatory cytokines and phagosome–lysosome fusion as well as the transcriptome of macrophages infected by ΔM5447 were analyzed to explore the role of PMT in host–pathogen interaction.

## Materials and Methods

### Bacterial Strains, Culture Media, and Plasmids

The wild-type *M. smegmatis* mc^2^155 strain (Wt), the *MSMEG*_5447 gene knockout strain (ΔM5447), and the *MSMEG*_5447 gene complemented strain (Comp) were cultured in liquid Middlebrook 7H9 broth containing 10% albumin–dextrose–catalase (ADC), 0.05% Tween 80, and 0.5% glycerol or in Middlebrook 7H11 solid medium supplemented with 10% ADC and 0.5% glycerol. These bacterial strains were also grown in LBT medium (LB broth containing 0.05% Tween 80) or LB agar. Kanamycin (25 μg/ml) and hygromycin (50 μg/ml) were used for the selection of appropriate strains. The *Escherichia coli*–*Mycobacterium* shuttle plasmid pMind (Blokpoel et al., 2005) was used to overexpress MSMEG_5447 protein in the ΔM5447 strain. The pCG76-GFP plasmid (Deng et al., 2016) was used to express green fluorescent protein (GFP) in different *M. smegmatis* strains.

### Construction of MSMEG_5447 Gene Complemented Strain (Comp)

The *M. smegmatis* mutant with *MSMEG_5447* disruption, ΔM5447, was constructed by using DNA homologous recombination in our previous studies (Deng et al., 2016). For constructing *MSMEG*_5447 gene-complemented strain (Comp), the *MSMEG_5447* gene (1551 bp) was amplified by PCR from *M. smegmatis* mc2155 genomic DNA using a forward primer (5′AGCATATGACCGCCCTCGACACCGATAC3′, underlined is the NdeI site) and a reverse primer (5′GTACTAGTCTAGTGATGATGGTGATGGTGGCGCCA-GCTCGGCAACC3′, underlined is the SpeI site). The PCR product of the *MSMEG_5447* gene was cloned to the pJET1.2/blunt vector, yielding a pJET-*MSMEG_5447* plasmid. After confirmation by DNA sequencing, the *MSMEG_5447* gene was inserted into expression vector pMind, thereby generating pMind-*MSMEG_5447* plasmid. The pMind-*MSMEG_5447* plasmid was transformed into ΔM5447 electro-competent cells, generating a *MSMEG_5447* gene complementary strain, Comp. The expression of His-tagged MSMEG_5447 protein was induced by tetracycline (20 ng/ml) for 24 h and detected by Western blot with α(anti)-polyhistidine clone HIS-1 (Sigma) followed by AP-conjugated goat anti-mouse IgG (Proteintech, Rosemont, IL, United States) and finally visualized by using NBT/BCIP solution.

### ConA Lectin Blot of Bacterial Proteins

Whole-cell lysate (WCL), CW, cell membrane (CM), and soluble (SOL) fractions were separated by differential ultracentrifugation as described previously (Gibbons et al., 2007). Briefly, 100 ml of bacterial cultures was harvested by centrifugation. The bacterial cells were resuspended in 5 ml of lysis buffer (PBS pH 7.4, 1 mM phenylmethylsulfonyl fluoride) and lysed by sonication. Lysates were centrifuged at 3,000 × *g* for 30 min to get WCL, which was centrifuged at 27,000 × *g* for 30 min to obtain the supernatant and CW pellet. The supernatant was then centrifuged at 100,000 × *g* for 2 h to acquire the CM pellet and SOL fraction. CW and CM fractions were resuspended in 0.5 ml of lysis buffer. The concentration of proteins was determined by BCA Protein Quantification kit (Vazyme, Nanjing, China). After separation by SDS-PAGE, the gels was stained with Coomassie Brilliant Blue R250 or transferred onto a nitrocellulose (NC) membrane for 1 h. The NC membrane was blocked with 3% BSA in TBST buffer for 2 h at room temperature and incubated with biotinylated ConA lectin (Vector Laboratories, Burlingame, CA, United States) at 1:10,000 dilution at 4°C overnight. After three times washing with TBST buffer, the NC membrane was incubated with streptavidin-HRP (Beyotime Biotechnology, Shanghai, China) at 1:20,000 dilutions for 1 h at room temperature. Finally, the protein bands were visualized by adding WesternBright ECL detection reagents (Advansta, Menlo Park, CA, United States).

### Macrophage Infection and Colony-Forming Units (CFU) Determination

THP-1 macrophage, a suspension cell line, was cultured in RPMI 1640 (Gibco) medium supplemented with 10% fetal bovine serum (FBS) (Lonsera) and penicillin–streptomycin (HyClone) solution in a 37°C incubator with 5% CO_2_. THP-1 cells were seeded into 12-well plates at a density of 5 × 10^5^ cells/well and differentiated into macrophages by inducing with 100 ng/ml phorbol 12-myristate 13-acetate (PMA) for 24 h. Prior to infection, the bacterial cultures of Wt, ΔM5447, and Comp were centrifuged at 3000 × *g* for 5 min and the pellets were resuspended in RPMI 1640 medium without antibiotic and FBS. The THP-1 cells were washed with PBS and infected by Wt, ΔM5447, or Comp at a multiplicity of infection (MOI) of 10 for 3 h. The THP-1 cells were washed with PBS three times to remove extracellular bacteria and then cultured in RPMI 1640 medium with 10% FBS and 50 μg/ml gentamicin for 1 h to completely remove the extracellular bacteria. This time point was regarded as 0 h of post-infection. After that, the cells were washed with PBS for three times and then cultured in RPMI 1640 medium with 10% FBS and penicillin–streptomycin for an additional 24 h. For the colony-forming unit (CFU) assay, the cells were lysed with 200 μl 0.03% SDS for 5 min. The lysates with 10-fold serial dilutions were plated on the LB agar plates, and the number of colonies was counted 2–3 days later. For cytokine detections, the cell culture was collected at 24 h post-infection and the production of cytokines was analyzed by ELISA.

### Immunofluorescence Assay

The pCG76-GFP vector was electro-transformed to Wt, ΔM5447, and Comp strains generating GFP-expressing Wt, ΔM5447, and Comp strains, respectively. THP-1 cells were seeded on glass slide in 24-well plates and stimulated with PMA (100 ng/ml) for 24 h. The THP-1 cells were infected with GFP-expressing Wt, ΔM5447, or Comp at an MOI of 10 for 3 h. The infected cells were cultured in RPMI 1640 medium with 10% FBS and 5 μg/ml gentamicin for 2 and 24 h. The cells were washed with PBS and fixed in 4% paraformaldehyde (PFA) for 20 min at room temperature. The cells were permeabilized in 0.2% Triton-X 100 for 5 min. For visualizing intracellular bacteria, F-actin of cells was stained with rhodamine phalloidin for 30 min in the dark. For detecting the expression of lysosomal-associated membrane protein 1 (LAMP-1) and NF-kB p65, cells were fixed in 4% PFA and incubated with blocking buffer (3% BSA) for 30 min. Then, the cells were incubated with anti-LAMP-1 (Abgent, San Diego, CA, United States) or anti-p65 (Proteintech, United States) antibody overnight at 4°C. Finally, the coverslids were incubated with secondary anti-rabbit antibodies conjugated to Alexa or FITC for 1 h at room temperature. For observing the co-localization of intracellular bacteria with lysosome, cells were incubated with 500 nM LysoTracker Red (Invitrogen) in RPMI 1640 medium for 30 min and fixed in 4% PFA for 20 min. Fluoroshield with DAPI (Abcam, Cambridge, MA, United States) was used for staining nucleic acid. The co-localization of LAMP-1 or lysosome with GFP-expressing bacteria was analyzed in more than 100 cells. Images were visualized with a fluorescence microscope (Olympus). Experiments were performed in two independent experiments.

### Resazurin Assay

Lysozyme resistance of bacteria was examined according to the published method (Palomino et al., 2002). The mycobacterial cell suspension was prepared by diluting the bacterial culture in LBT broth at 1:5,000, and its accurate density was confirmed by CFU counting on LB agar plates. The mycobacterial cell suspension of 50 μl was added into 96-well plates followed by adding 50 μl of lysozyme with two-fold serial dilutions. The well containing bacteria without lysozyme were regarded as control. After incubation for 24 h in a 37°C incubator, 100 μl resazurin solution (1:1 mixture of 125 μg/ml resazurin and 10% Tween 80) was added to each well and the plate was incubated for an additional 5–12 h for color development. The blue color of resazurin dye changes to pink in the reducing environment of living cells.

### The Acidic Stress Assay

The LBT medium was prepared and adjusted to pH 5.0 by adding HCl before sterilization by filtration using a 0.22-μm filter. The bacterial cultures were diluted to an OD_600 *nm*_ of 0.5 in LBT medium and added into acidic broth at 1:100 dilutions. After incubation for 0, 12, 24, and 36 h, the viability of bacteria was determined by plating bacteria at 10-fold serial dilutions on LB agar plates and counting bacterial CFU 2–3 days later. The growth of bacteria was also monitored by measuring OD_600 *nm*_ at an interval of 12 h after exposure to acidic broth.

### Ethidium Bromide Accumulation Assay

Strains of Wt, ΔM5447, and Comp were grown in LBT medium to an OD_600 *nm*_ of 1.0. Cultures were washed and resuspended with PBS containing 0.05% Tween 80. The OD_600 *nm*_ of bacterial suspension was adjusted to 0.5 with PBST, and 200 μl suspension was added to a 96-well black fluoroplate with three replicates. Ethidium bromide (EB) at concentrations of 2 μg/ml was added. The EB accumulation of strains was measured in the BioTek Synergy NEO with an excitation of 544 nm and emission of 590 nm. Fluorescence data was acquired for 1 h at an interval of 5 min. All data from each well were normalized to the time of zero reading. All experiments were repeated two times, and similar results were obtained.

### Quantitative Real-Time PCR (qPCR)

THP-1 cells were infected with Wt, ΔM5447, or Comp strain for 3 h and cultured for an additional 24 h. The infected cells were harvested, and their total RNA was extracted using RNAiso Plus (Takara, Mountain View, CA, United States) reagent according to the manufacturer’s protocol. The cDNA was synthesized with reverse-transcription of RNA (1 μg) by using the PrimeScript RT Reagent Kit with genomic DNA Eraser (Takara). The cDNA of 20 ng served as a template for quantitative real-time PCR (qPCR). The reaction was performed in StepOnePlus Real-Time PCR System (Applied Biosystems, Foster City, CA, United States) using SYBR Green Premix Ex Taq II (Takara) and gene-specific primers (Table 1). The amplification condition was as follows: 30 s at 95°C for initial denaturation, and 5 s at 95°C and 30 s at 60°C for 40 cycles. The melting curve was used to confirm the specificity of primers. All qPCR reactions were performed for three independent experiments, and the relative expression of specific genes was evaluated using the 2^–ΔΔCT^ method.

**TABLE 1:** Primers used in qPCR reactions.

**Genes**	**Primer sequences (5′–3′)**
TNF-α	Forward: GCTGCACTTTGGAGTGATCG
	Reverse: ACATGGGCTACAGGCTTGTC
IL-6	Forward: ACTCACCTCTTCAGAACGAATTG
	Reverse: CCATCTTTGGAAGGTTCAGGTTG
NF-κB	Forward: ATGGAGAGTTGCTACAACCCA
	Reverse: CTGTTCCACGATCACCAGGTA
GAPDH	Forward: AGCCTCAAGATCATCAGCAATG
	Reverse: TGTGGTCATGAGTCCTTCCACG
	

### Cytokine Measurement

The culture supernatant of infected cells was collected at 24 h post-infection. The concentrations of tumor necrosis factor α (TNF-α), interleukins (IL)-6, IL-12, and IL-10 were measured by ELISA kits (Xinfan Biological Company, Shanghai, China) following the manufacturer’s instructions.

### Protein Preparation and Western Blot Analysis

The total proteins of THP-1 cells infected with Wt, ΔM5447, or Comp strain were prepared at 24 h post-infection, and the concentration of proteins was determined by a BCA Protein Quantification kit (Vazyme). After separation of proteins by SDS-PAGE, the proteins were transferred onto an NC membrane. The membrane was blocked with 5% (w/v) non-fat milk in TBST buffer for 2 h at room temperature and incubated with primary antibodies, NF-κB p65 (Cell Signal Technology, Danvers, MA, United States), pSer536-NF-κB p65 (Cell Signal Technology), or GAPDH (Proteintech), at 4°C overnight. After washing three times with TBST buffer, the membrane was incubated with appropriate HRP-conjugated secondary antibody (Beyotime Biotechnology) for 1 h at room temperature. Finally, the protein bands were visualized by Western Bright ECL detection reagents (Advansta) and quantified using ImageJ software.

### Transcriptomic Analysis of Infected THP-1 Cells by RNA Sequencing

For RNA sequencing, total RNA was extracted from THP-1 cells infected with Wt and ΔM5447 strain at 24 h post-infection. Each group was performed in duplicate. The RNA quantity and quality were evaluated using the Agilent 2100 bioanalyzer and agarose gel electrophoresis. The cDNA libraries were constructed using NEB Next Ultra^TM^ RNA Library Prep Kit for Illumina (NEB, Ipswich, MA, United States), and the libraries were sequenced on the Illumina HiSeq platform by Novogene Bioinformatics Technology (Beijing, China). Clean data were obtained by removing linker sequences and low-quality bases from raw data. The clean reads were evaluated by Q20, Q30, and GC contents and mapped to the human transcriptome (RefSeq transcriptome index hg19). For analyzing gene expression profiles, differentially expressed genes (DEGs) of two groups were identified by using the DESeq2 R package and an adjusted *p*-value < 0.05 as the threshold criteria. For functional annotation, gene ontology (GO) enrichment analysis of DEGs was implemented by the cluster Profiler R package and the GO terms with corrected *p*-value less than 0.05 were considered significantly enriched. Kyoto Encyclopedia of Genes and Genomes (KEGG) pathway analysis of DEGs was done in the KEGG database^1^. Interactions of key genes that presented in two enriched pathways were analyzed by STRING and shown by Cytoscape software. The raw data of RNA-Seq with accession number GSE128970 are available at the NCBI GEO database (Edgar et al., 2002).

### Statistical Analysis

The statistical analysis of data was performed by using GraphPad Prism 8.0. Comparisons between groups were conducted by the method of Student’s unpaired *t*-test or two-way ANOVA. Data were shown as means ± SD. *p* < 0.05 was considered as statistical significance.

## Results

### Inactivation of MSMEG_5447 Impaired *M. smegmatis* Growth and Its Protein O-Mannosylation

**FIGURE 2:**
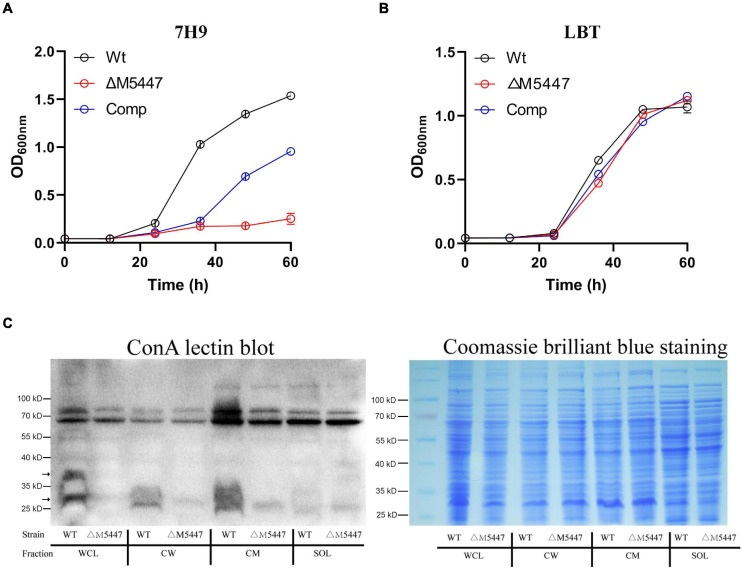
Protein O-mannosyltransferase (PMT) deficiency impaired the growth of *M. smegmatis* and reduced its level of O-mannosylation. Wild-type *M. smegmatis* mc^2^155 (Wt) strain and MSMEG_5447 gene knockout strain (ΔM5447) and Comp strain were grown in LBT medium **(A)** or Middlebrook 7H9 medium supplemented with 10% ADC and 0.05% Tween 80 **(B)**; the growth rate of bacteria was evaluated by measuring OD_600_ at an interval of 12 h. **(C)** Wt and ΔM5447 strains were grown in LBT broth. After the bacterial pellet was lysed by sonication, whole-cell lysate (WCL), CW, CM, and soluble (SOL) fractions were separated by differential centrifugation. The level of O-mannosylation of proteins in each subcellular fraction was analyzed by ConA lectin blotting (Left). The gels stained with Coomassie brilliant blue showed the same loading amount between different samples (Right). The black arrows for the band indicate the different levels of O-mannosylation in fractions of Wt and ΔM5447 strains.

To characterize the impact of PMT on physiology of the role of *M. smegmatis*, *MSMEG_5447* gene-complemented strain, Comp, was constructed (Supplementary Figure 1). The growth of Wt, ΔM5447, and Comp strain was measured in Middlebrook 7H9 broth and LBT medium. Our data showed that the growth rate of the ΔM5447 strain was significantly reduced in 7H9 broth (Figure 2A) and the colonies of ΔM5447 were small and loose as compared to that of Wt (Supplementary Figure 2). Interestingly, ΔM5447 had a similar growth rate to the Wt and Comp strains in LBT medium (Figure 2B). Subsequently, O-mannosylation of proteins in different subcellular fractions was evaluated by differential centrifugation followed by ConA lectin blot. As shown in Figure 2C, the level of O-mannosylation of protein at 25–40 kD in CW and CM fractions of ΔM5447 was reduced as compared to that of the Wt strain when the same amount of samples was observed in Coomassie brilliant blue staining gel. We also found that the level of O-mannosylation of proteins around 70 kD had no differences in all fractions except CM of the Wt and ΔM5447 strains. One possible explanation is that the bands around 70 kD might derive from recognition of α-glucose as ConA can recognize both α-mannose and α-glucose. Furthermore, analysis of O-mannosylation in the supernatant and pellet also indicated that O-mannosylation of proteins in the ΔM5447 strain was decreased as compared to that of the Wt and Comp strains (Supplementary Figure 3).

### The Lysosomal Resistance of ΔM5447 Strain was Impaired

**FIGURE 3:**
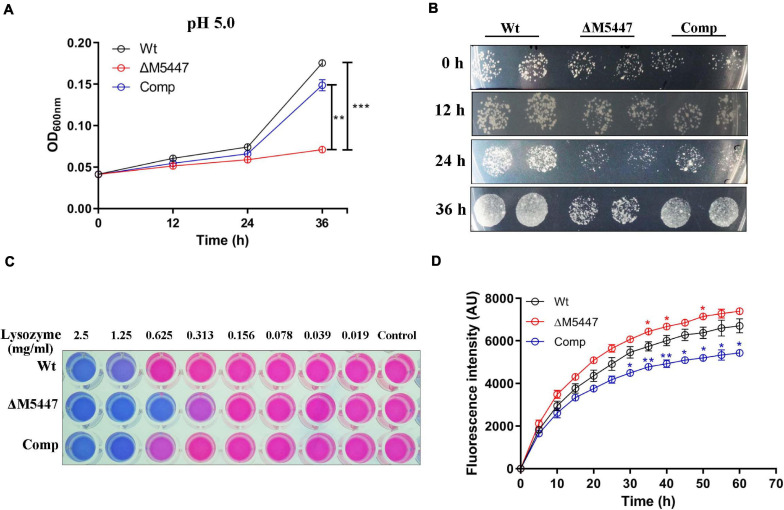
The tolerance of ΔM5447 to lysosome-related stress was reduced. **(A)** Wt, ΔM5447, and Comp strains were cultured in LBT acidic medium (pH 5.0); the growth of bacteria was determined by measuring OD_600_ at an interval of 12 h. **(B)** The corresponding cultures from acidic medium were plated on LB agar plates with a 100-fold dilution at an interval of 12 h. **(C)** Wt, ΔM5447, and Comp strains were treated with lysozyme in a two-fold serial dilution for 24 h. Resazurin dye was added and incubated for 5–12 h for color development. The changed color from original blue to pink indicated that the living cells existed. The well contained bacterial culture only as control. A representative result was shown from three independent experiments. **(D)** Mid-log phage cultures of Wt, ΔM5447, and Comp strains were incubated in PBST with 2 μg/ml EB. The EB accumulation of strains was observed for 1 h at an interval of 5 min. The value at each point is normalized to the time of zero value. Data were shown as mean ± SD of triplicate wells. Statistical analyses were performed by the method of two-way ANOVA (*p < 0.05, **p < 0.01, ***p < 0.001).

Exposure of bacteria in an acidic condition, *in vitro*, has been specifically used to mimic the bacteria in acidic phagolysosome, which was considered as a key strategy for the host to clear bacteria during infection (Vandal et al., 2009). Therefore, the effect of PMT deficiency on bacterial resistance to lysosome-related stress was assessed *in vitro* in the LBT medium. The growth of Wt, ΔM5447, and Comp strains in acidic LBT medium (pH 5.0) was examined by monitoring OD_600_ and counting bacterial CFU. As shown in Figure 3A, the growth of ΔM5447 in acidic LBT medium was significantly reduced compared to that of Wt and Comp strains after 24 and 36 h of incubation. The CFU results showed that the ΔM5447 strain had a lower rate of viability in acidic culture than those of Wt and Comp strains after 36 h of incubation (Figure 3B). The viability of Wt, ΔM5447, and Comp strains under lysozyme stress was also determined by resazurin microplate assay. *M. smegmatis* ΔM5447 showed lower resistance to lysozyme (MIC = 313 μg/ml) as compared to that of the Wt strain and Comp strain (MIC = 625 μg/ml) (Figure 3C). The CW permeability was evaluated by measuring the EB accumulation in Wt, ΔM5447, and Comp strains. The results showed that EB accumulation was significantly increased in ΔM5447 as compared with Wt strain, and that increase in ΔM5447 was reversed in Comp strain (Figure 3D).

### The Viability of ΔM5447 in THP-1 Macrophages Was Reduced

**FIGURE 4:**
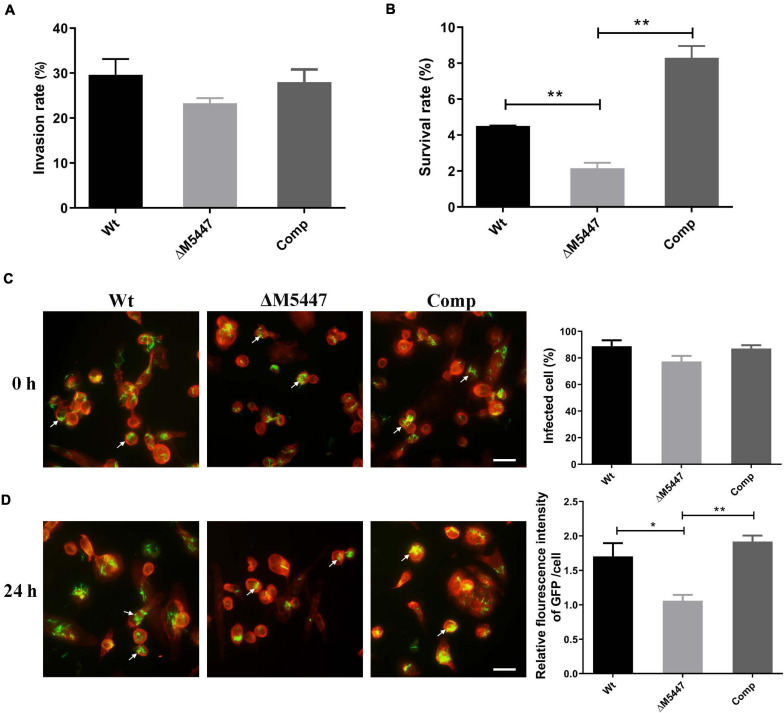
ΔM5447 displayed impairment in survival ability in THP-1 cells. The invasion ability **(A)** and intracellular survival **(B)** of different *M. smegmatis* strains in THP-1 macrophage were evaluated. Macrophage cells were infected with Wt, ΔM5447, and Comp strains at an MOI of 10. The infected cells were lysed by 0.03% SDS at the indicated time, and the colonies were counted by CFU assay. The invasion rate of bacteria was calculated by the number of intracellular bacteria at 0 h post-infection to the number of initial bacteria for infection. The percentage of intracellular survival of bacteria was evaluated by the intracellular bacteria at 24 h post-infection to the number of 0 h of post-infection. The invasion and survival ability of bacteria in THP-1 macrophages were also determined by fluorescence microscopy. Green fluorescence protein (GFP)-expressing plasmid and rhodamine-phalloidin dye were used to visualize the bacteria and macrophage F-actin individually. **(C)** The bacterial invasion rate was evaluated by the percentage of cells containing GFP at 0 h post-infection. **(D)** The bacterial survival rate was evaluated by the relative fluorescence intensity of GFP per cell at 24 h post-infection. The white arrows indicate a representative number of intracellular bacteria. Scale bars, 20 μm. A representative field was shown from two independent experiments. Data were shown as mean ± SD of replicate wells; statistical analyses were performed by the method of two-tailed *t*-test (*p < 0.05, **p < 0.01).

To evaluate the impact of PMT deficiency on host–pathogen interaction, THP-1 macrophage cells were infected with Wt, ΔM5447, or Comp strain for 3 h and the invasion rate and subsequent intracellular survival of bacteria at 24 h post-infection was examined by CFU assay. As shown in Figure 4A, the invasion rate of ΔM5447 strain exhibited a slight but not significant reduction as compared to that of the Wt strain at 0 h post-infection. However, inside THP-1 cells, the survival of ΔM5447 was significantly reduced as compared to that of the Wt strain at 24 h post-infection, and this decline was reversed in the Comp strain (Figure 4B). To further confirm the above results, GFP-expressing bacteria and F-actin of macrophages were visualized by fluorescence microscopy. As shown in Figure 4C and Supplementary Figure 4, the percentage of infected THP-1 cells had no significant difference among Wt, ΔM5447, and Comp strains at 0 h post-infection. However, 24 h after infection, the relative fluorescence intensity of GFP was significantly reduced in ΔM5447-infected THP-1 cells as compared to the Wt- and Comp-infected cells (Figure 4D). These data indicated that PMT inactivation decreased the survival of *M. smegmatis* in macrophages.

### ΔM5447 Infection Impaired the Production of TNF-α and IL-6 of Macrophage

**FIGURE 5:**
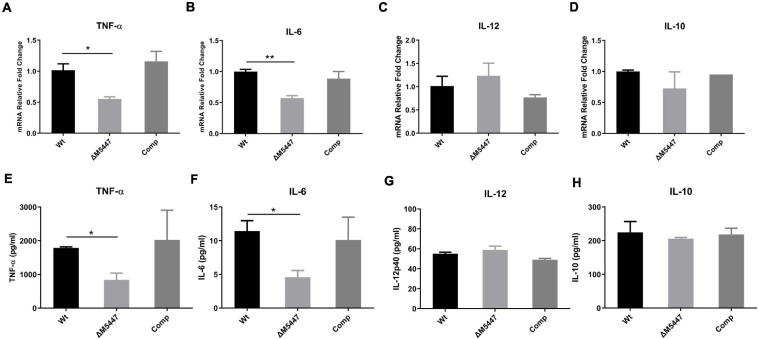
ΔM5447 strain decreased the production of pro-inflammatory cytokines in macrophage. THP-1 macrophages were infected with Wt, ΔM5447, and Comp strains for 3 h at a MOI of 10. Total RNA of infected cells was extracted at 24 h post-infection, and the transcription levels of TNF-α **(A)**, IL-6 **(B)**, IL-12p40 **(C)**, and IL-10 **(D)** were measured by qPCR. Their mRNA levels were normalized by GAPDH mRNA. Culture supernatants were collected, and the production of TNF-α **(E)**, IL-6 **(F)** IL-12p40 **(G)**, and IL-10 **(H)** was evaluated by ELISA. Data were shown as mean ± SD of triplicate wells. Statistical analyses were performed by the method of two-tailed *t*-test (*p < 0.05, **p < 0.01).

To explore whether PMT deficiency in mycobacteria could affect the inflammatory response of infected macrophages, the production of cytokines was evaluated by qPCR and ELISA. We found that the expression of TNF-α (Figure 5A) and IL-6 (Figure 5B) was significantly reduced in ΔM5447-infected THP-1 cells as compared to Wt-infected THP-1 cells at the transcriptional level. Consistently, ELISA results showed that the secretion of TNF-α and IL-6 was significantly decreased in ΔM5447-infected THP-1 cells (Figures 5E,F). There was no difference on the transcription and secretion of IL-10 and IL-12 when THP-1 cells were infected with Wt, ΔM5447, or Comp strain (Figures 5C,D,G,H).

### ΔM5447 Infection Reduced the Expression and Activation of NF-κB in Macrophage

**FIGURE 6:**
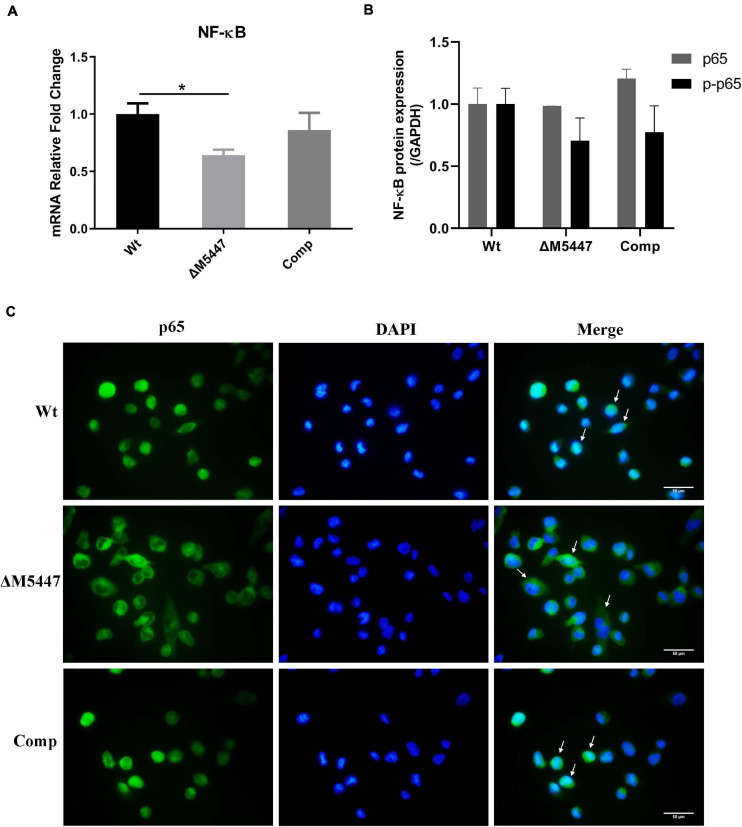
The expression and activation of NF-κB were decreased in ΔM5447-infected macrophages. THP-1 macrophage cells were infected with Wt, ΔM5447, and Comp strains for 3 h. **(A)** The total RNA was extracted from infected THP-1 cells at 24 h post-infection, and the transcriptional level of the p65 subunit of NF-κB was evaluated by qPCR. **(B)** The total protein was extracted from infected THP-1 cells at 24 h post-infection, and the protein levels of p65 and p-p65 were determined by western blot. Densitometric analyses of images of WB have been presented. **(C)** The translocation of the p65 subunit of NF-κB from cytosol to nucleus was evaluated by fluorescence microscopy at 24 h post-infection. The cells were fixed and stained with p65 antibody and DAPI. The white arrows indicate the translocation of the p65 subunit of NF-κB. Scale bars, 50 μm. Data were shown in representative results from three independent experiments. Statistical analyses were performed by the method of two-tailed *t*-test (*p < 0.05).

As a downstream executor of the signal transduction pathway, nuclear factor NF-κB can be activated and thus increase the expression of inflammatory-related genes (Pahl, 1999). To determine whether NF-κB was affected in ΔM5447-infected macrophages, the expression and activation of NF-κB were evaluated in macrophages at 24 h after infection with Wt, ΔM5447, or Comp strain. The qPCR result showed that NF-κB was down-regulated by 35% in ΔM5447-infected macrophages as compared to Wt-infected macrophages, which was partially reversed in Comp-infected macrophages (Figure 6A). Western blot results showed that the phosphorylation of p65 (p-p65) was reduced in ΔM5447-infected macrophages as compared to the Wt-infected macrophages (Figure 6B). Furthermore, the activation of NF-κB was evaluated by detecting the translocation of the p65 subunit to the nucleus. At 24 h infection, the nuclear translocation of the p65 subunit was reduced in ΔM5447-infected macrophages as compared to the Wt or Comp-infected macrophages (Figure 6C). Taken together, our data indicated that PMT deficiency impaired the inflammatory response of macrophages stimulated by mycobacteria.

### ΔM5447 Strain Enhanced the Phagolysosomal Fusion in Macrophages

**FIGURE 7:**
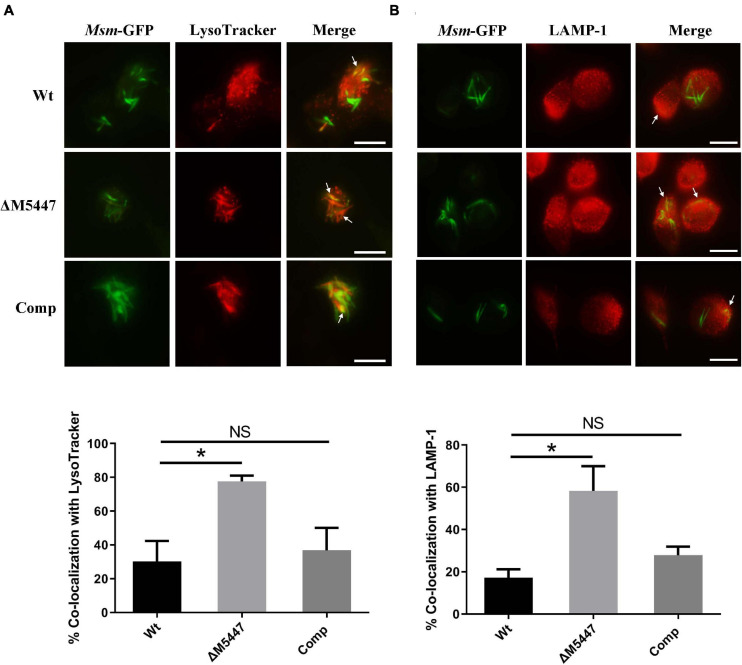
ΔM5447 stain failed to arrest the phagosome–lysosome fusion in infected macrophages. THP-1 macrophages were infected with green fluorescence protein (GFP)-expressing Wt, ΔM5447, and Comp strains for 3 h at an MOI of 10. After 24 h of infection, cells were stained with LysoTracker Red for 30 min before fixation **(A)** or incubated with a LAMP-1 antibody **(B)**. The cells were visualized by fluorescence microscopy. The yellow color represented the co-localization of green and red fluorescence, as shown in images. The white arrows indicate the co-localization of green and red fluorescence. Scale bars, 25 μm. Quantitation of co-localization was shown as mean ± SD in at least 100 random infected cells. NS, there was no statistically significant difference between groups. Statistical analyses were performed by the method of two-tailed *t*-test (*p < 0.05).

Inhibition of phagosome–lysosome fusion is another effective strategy used by Mtb to evade microbicidal activity of macrophages (Carranza and Chavez-Galan, 2018). We proposed that the reduced survival of ΔM5447 in macrophages was due to the failure of blocking phagosome–lysosome fusion. To test this possibility, THP-1 macrophages were infected with GFP-expressing Wt, ΔM5447, or Comp strain and then stained with LysoTracker Red which is a red-fluorescent dye to label acidic lysosomes. The phagosome–lysosome fusion was assessed by co-localization of lysosome with intracellular GFP-expressing bacterial strains. As shown in Figure 7A and Supplementary Figure 5A, Wt displayed 30% co-localization with LysoTracker Red whereas ΔM5447 showed 77% co-localization (*p* < 0.05). Comp showed 36% co-localization with lysosome which was similar with Wt. To further assess the effect of MSMEG_5447 on phagosome–lysosome fusion, co-localization of intracellular GFP-expressing bacteria with LAMP-1 was observed under a fluorescence microscope. LAMP-1 is delivered to phagosomes during the phagosome maturation process and considered as a late endosomal-lysosomal marker. As shown in Figure 7B and Supplementary Figure 5B, Wt showed 17% co-localization with LAMP-1 whereas ΔM5447 showed 58% co-localization (*p* < 0.05). Comp showed 27% co-localization with LAMP-1, which was not significantly different from Wt. In addition, the expression of MR was observed in mycobacteria-infected cells at 0 h of post-infection by flow cytometry in our preliminary expreiment, showing that the expression of MR in ΔM5447-infected cells was decreased as compared to that of the Wt and Comp strains (Supplementary Figure 6). These data indicated that PMT deficiency enhanced phagosomal maturation in macrophages.

### Transcriptome of ΔM5447-Infected Macrophages Was Analyzed

**FIGURE 8:**
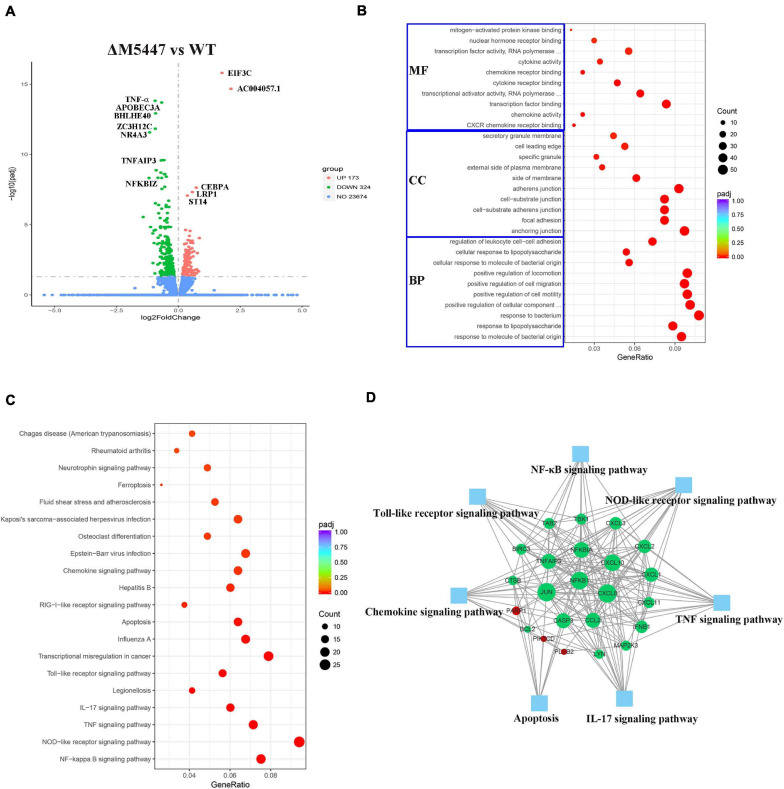
Transcriptional profiles of macrophages during mycobacterial infection. THP-1 macrophage cells were infected with Wt and ΔM5447 strains for 3 h, and the total RNA of THP-1 cells was extracted at 24 h post-infection. **(A)** Volcano plots of differentially expressed genes (DEGs) in ΔM5447-infected THP-1 cells as compared to the Wt-infected THP-1 cells. The name of DEGs was annotated next to the dots. Blue, green, and red splashes represent genes without significant change, significant down-regulation, and significant up-regulation, respectively. **(B)** Scatter plot of GO functional classification of the DEGS. The distributions are summarized in three main categories: biological process (BP), molecular function (MF), and cellular component (CC). GeneRatio is the ratio of the DEG number to the total gene number in each category. The color and size of the dot represent the range of the adjusted *p*-value and the number of DEGs mapped to the categories. The top 10 functions in each category are shown in the figure. **(C)** Scatter plot of enriched KEGG pathways. GeneRatio is the ratio of the DEG number to the total gene number in a certain pathway. The color and size of the dot represent the range of the adjusted *p*-value and the number of DEGs mapped to the indicated pathways, respectively. The top 20 enriched pathways are shown in the data. **(D)** Interaction of key genes that presented in two pathways at least were analyzed by STRING and shown by Cytoscape software. Green and red nodes represent the down- and up-regulated genes, respectively. The size of the node indicated the core role of the gene. Blue squares represent the KEGG pathways.

RNA sequencing is a powerful tool in analyzing the gene expression pattern of cells under specific physiological conditions (Nalpas et al., 2015). To further confirm the effect of PMT deficiency of *M. smegmatis* on macrophage infection, the transcriptome of THP-1 cells infected with ΔM5447 or Wt strain was analyzed by RNA sequencing at 24 h post-infection. As shown in a Volcano plot, in total, 497 DEGs were identified in ΔM5447-infected THP-1 cells using adjusted *p*-value < 0.05 as the threshold criteria as compared to the Wt-infected THP-1 cells (Figure 8A and Supplementary Table 1). Among them, 173 genes were up-regulated and 324 genes were down-regulated. ACOO4057 and EIF3C involved in transcription and translation processes were most significantly up-regulated. The expressions of TNF-α, NF-κB (NF-κB 1, NF-κB 2, NF-κB IA), and IFN-β were down-regulated in ΔM5447-infected THP-1 cells. TNF-α was most significantly down-regulated in all down-regulated genes. In addition, chemokines CXCL1, 2, 3, 8, 10, 11, and 12 were also significantly down-regulated. To better understand the effect of the ΔM5447 strain on macrophages, all DEGs were further mapped to GO and KEGG databases. Totally, 464 out of 497 DEGs were assigned to 841 GO terms, including 788 biological processes (BP), 25 cellular components (CC), and 28 molecular function (MF) terms. Typical GO terms are shown in Figure 8B. Most of the BP categories were related to “response to bacterium” and “positive regulation of CC movement.” The two highest percentages of GO terms under the CC category were “anchoring junction” and “adherens junction.” The mainly enriched MF categories were “Transcription factor binding” and “transcriptional activator activity.” The top 20 enriched KEGG pathways are shown in Figure 8C, and some of them were involved in inflammatory response such as NF-κB pathway, TNF signaling pathway, NOD-like receptor signaling pathway, IL-17 signaling pathway, and Toll-like receptor signaling pathway. The interaction of proteins that were enriched in at least two pathways was analyzed using STRING. As shown in Figure 8D, among 23 genes, NF-κB, JUN, and CXCL8 played a core role in enriched pathways.

Overall, these results demonstrated that PMT deficiency reduced the intracellular survival of *M. smegmatis*, which was associated with failure of inhibiting the phagosome–lysosome fusion in macrophages. Meanwhile, lacking PMT also impaired the capability of *M. smegmatis* to stimulate inflammatory response in macrophages.

## Discussion

Protein O-mannosylation in Mtb is frequently found in virulence-related secreted and cell-wall lipoproteins, which plays a crucial role in Mtb pathogenicity (Birhanu et al., 2019). Despite identification of Rv1002c as PMT in Mtb and demonstration of its vital importance for the Mtb interaction with the host, the current knowledge about this enzyme remains limited and its characteristics in the process of infection have not been fully elucidated so far. Previously, we found that the *M. smegmatis MSMEG_5447* gene knockout strain (ΔM5447) failed to produce mannosylated protein Rv0431 (Deng et al., 2016). We supposed that the lack of PMT would exert profound impacts on mycobacterial interaction with host innate immune responses by regulating the O-mannosylation of proteins. In this study, the ΔM5447 strain was utilized and its complementary stain (Comp) was generated. We found that the ΔM5447 strain displayed a lower level of O-mannosylation of proteins and decreased resistance to lysozyme and acidic medium. We also found that the survival of ΔM5447 in THP-1 macrophage cells was impaired and its mechanism related to the failure of inhibition for phagosome–lysosome fusion.

*Mycobacterium smegmatis*, a non-pathogenic mycobacterium, has been widely used as a tool for the study of many aspects of mycobacterial infections. To clarify the function of proteins, it is often used as an alternative host to express proteins of pathogenic mycobacteria (Bashiri and Baker, 2015). Additionally, the results from Diaz-Silvestre et al. (2005) had shown that *M. smegmatis* could express Mtb 19-kDa antigen which was a glycoprotein with O-mannosylation modification and promoted bacterial adhesion to the macrophage *via* mannosyl residues. It indicated that *M. smegmatis* has the same glycosylation system as Mtb and can also be used to investigate recognition and interaction with the host cells. Furthermore, PMT in *M. smegmatis* shared 75% similarity with Mtb and have conserved residues in active sites. In our study, we found that the growth of ΔM5447 was different in LBT and 7H9 broth. We speculated that different growth patterns of the ΔM5447 strain may be due to different nutritional components. As Liu et al. reported, the Rv1002c mutant was almost completely unable to grow in 7H9 medium supplemented with dextrose only but just displayed a slight growth delay in ADC-enriched 7H9 medium. In order to eliminate the difference of the growth rate, the Wt, ΔM5447, and Comp strains were cultured in LBT medium in the following investigations. Previous studies have reported that many glycoproteins and lipoglycoproteins are either CW-associated or surface-exported proteins and depend on sec-translocation. Indeed, in our study, the loss of PMT dramatically reduced the level of O-mannosylation of proteins with 25–40 kD in CW and CM fractions of *M. smegmatis* (Birhanu et al., 2019). Next, we demonstrated that O-mannosyltransferase is dispensable for *M. smegmatis* survival during infection of macrophages since the intracellular survival of ΔM5447 was impaired during THP-1 infection. This finding is consistent with the reports that inactivation of Rv1002c largely reduced the intracellular survival of Mtb in THP-1 macrophage cells (Liu et al., 2013). In our study, the phagocytosis rate between WT and PMT-deficient *M. smegmatis* was similar. We speculated that it was due to the combined effect of pathogen-associated molecular pattern recognition receptors. The lack of O-mannosylation in mycobacteria affects not only the recognition between O-mannose residues with MR but also the recognition between glycoproteins and their corresponding receptors. Several studies revealed that some glycoproteins recognize TLR2 which also influenced the phagocytosis (Jung et al., 2006; Pecora et al., 2006; Drage et al., 2010). Additionally, it has been reported that the lack of O-mannosylation can affect the localization of proteins, and thus the change of localization of proteins will affect the phagocytosis (VanderVen et al., 2005; Arya et al., 2013).

Generally, as soon as Mtb enters into host cells, it will be exposed to various environmental or physiological stresses such as reactive oxygen or nitrogen intermediates, low pH, hypoxia, and alveolar surfactant (Manganelli et al., 2004). Here, our results demonstrated that the tolerance of ΔM5447 to lysozyme and acidic condition was significantly reduced. It has been reported that PMT deficiency increased CW permeability of *Mycobacterium abscessus* leading to the reduction in anti-tuberculosis drugs and lysozyme tolerance (Becker et al., 2017). Consistently, CW permeability of ΔM5447 was also increased in our study, indicating that the tolerant impairment of ΔM5447 might due to the increased CW permeability.

Macrophages play an essential role in the recognition, digestion, and degradation of invading pathogens (Queval et al., 2017). Mtb could utilize multiple strategies to survive and replicate within macrophages such as evasion of recognition and phagocytosis, attenuation of macrophage antigen presentation, interference with vesicular membrane trafficking, and manipulation of innate immune responses (Liu et al., 2017). By arresting the phagosome–lysosome fusion, Mtb could evade degradation and survive in macrophages, interfering cell immune defense (Carranza and Chavez-Galan, 2018). For example, protein tyrosine phosphatase PtpA secreted by Mtb could dephosphorylate the protein VPS33B of infected macrophages, leading to an inhibition of phagosome–lysosome fusion (Bach et al., 2008). In our study, we found that PMT could inhibit phagosome maturation as the phagosome containing ΔM5447 increased the co-localization with lysosome and LAMP-1. Our preliminary data showed that the expression of MR was reduced in ΔM5447-infected cells. Previous studies reported that glycolipoproteins Pst-S1 and LpqH as adhesin bound to the MR receptor promoting phagocytosis (Diaz-Silvestre et al., 2005; Esparza et al., 2015). Additionally, MR activation by glycopeptidolipids and ManLAM of Mtb was associated with arresting phagosome–lysosome fusion (Kang et al., 2005; Sweet et al., 2010). Therefore, we speculate that PMT inhibits phagosome–lysosome fusion in a mannose receptor (MR)-dependent manner.

Alonso et al. (2017) proposed that induction of pro-inflammatory response was partially responsible for the impaired survival of the Mtb PMT mutant in macrophages. Interestingly, our qPCR and ELISA data showed that inactivation of PMT in *M. smegmatis* decreased the production of IL-6 and TNF-α in macrophages. Our data also showed that ΔM5447 decreased the translocation of the p65 subunit to the nucleus and reduced the activation of NF-κB in macrophages. Consistently, our transcriptomic analyses revealed that inflammatory response-related pathways were significantly down-regulated in ΔM5447-infected macrophages. NF-κB plays a crucial role in inflammatory responses, immunity, apoptosis, and host defense, and its activation can induce a large number of inflammatory genes such as iNOS, TNF-α, and IL-6 (Pahl, 1999; Chen et al., 2017). We considered that production of pro-inflammatory cytokines was reduced in an NF-κB-dependent manner in ΔM5447-infected macrophages. The reduction of pro-inflammatory cytokines may be because of loss of protein O-mannosylation, affecting the TLR2-mediated pro-inflammatory response. It was reported that many glycolipoproteins were ligands of TLR2, such as LpqH, LprG, PstS-1, MPT83, and LprA (Noss et al., 2001; Gehring et al., 2004; Pecora et al., 2006; Sanchez et al., 2009; Wang et al., 2017). Based on the data we got so far, we are not clear whether the reduced pro-inflammatory response caused impairment of intracellular survival or not. The role of NF-κB in host defense may depend on both microbial features and host species. For example, Bai et al. (2013) have reported that inhibition of NF-κB activation decreased the intracellular survival of the mycobacterium in macrophage by promoting the apoptosis and autophagy in THP-1 cells.

In summary, our studies revealed that PMT was necessary for *M. smegmatis* survival within the macrophage and played a key role in arresting the phagosome maturation. We also found that the ΔM5447 mutant failed to activate the NF-κB pathway and subsequent pro-inflammatory response in macrophages. It is worth mentioning that PMT increased the tolerance of *M. smegmatis* to acidic stress *in vitro*. It makes us speculate that the protein O-mannosylation may also contribute to the survival of *M. smegmatis* in acidic phagolysosome pH. More detailed studies to clarify this mechanism are still needed. Since PMT is a PMT controlling the level of protein O-mannosylation, our study provides a new perspective to understand the impact of O-mannosylation of proteins in host–pathogen interaction.

## Data Availability Statement

The datasets presented in this study can be found in online repositories. The names of the repository/repositories and accession number(s) can be found below: https://www.ncbi.nlm.nih.gov/geo/, GSE128970.

## Author Contributions

YM, LJ, and SS designed the experiments, interpreted the results, and wrote the manuscript. LJ, SY, and AT performed the experiments and acquired and analyzed the data. All authors reviewed and discussed the manuscript.

## Conflict of Interest

The authors declare that the research was conducted in the absence of any commercial or financial relationships that could be construed as a potential conflict of interest.

## Footnotes

1 http://www.genome.jp/kegg/

## Abbreviations

ConA: concanavalin A
CW: cell wall
WCL: whole-cell lysate
CM: cell membrane
SOL: soluble fraction
CFU: colony-forming units
MOI: multiplicity of infection
PMA: phorbol 12-myristate 13-acetate
LBT: LB medium supplemented with 0.05% Tween 80
ADC: albumin–dextrose–catalase
LAMP-1: lysosome-associated membrane protein 1
DEGs: differentially expressed genes
PMT: protein O-mannosyltransferase
PSP-A: pulmonary C-type lectin surfactant protein A
Apa: alanine and proline-rich secreted antigen.
