# Supplementary material for: Effect of Protein O-Mannosyltransferase (MSMEG_5447) on M. smegmatis and Its Survival in Macrophages
Source: Front Microbiol. 2021 Jun 30;12:657726. doi: 10.3389/fmicb.2021.657726 (PMC8278756; doi:10.3389/fmicb.2021.657726)
Supplement: Supplementary file 1 [file Data_Sheet_1.PDF]

Supplementary Material

1. Supplementary Figures and Tables

1.1 Supplementary Figures

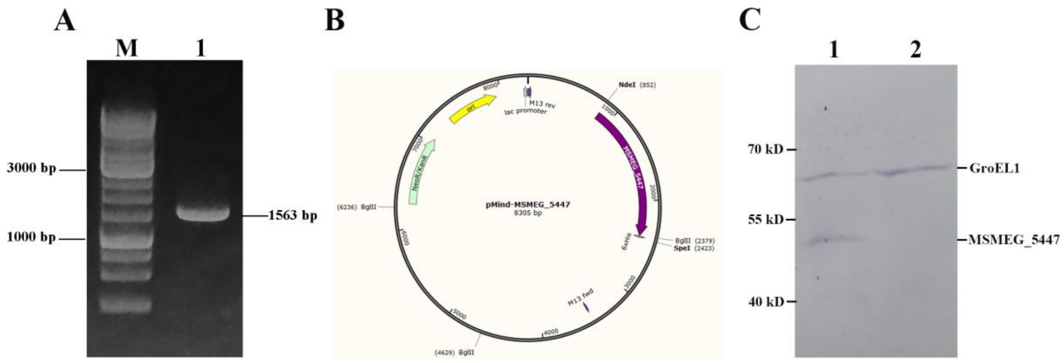

Figure S1. Construction of MSMEG_5447 gene complemented strain Comp. (A) PCR product of MSMEG_5447 gene; **(B)** Plasmid map of pMind-MSMEG_5447. **(C)** Expression of MSMEG_5447 protein in ΔM5447 strain was detected by Western blot with anti-His tag antibody.

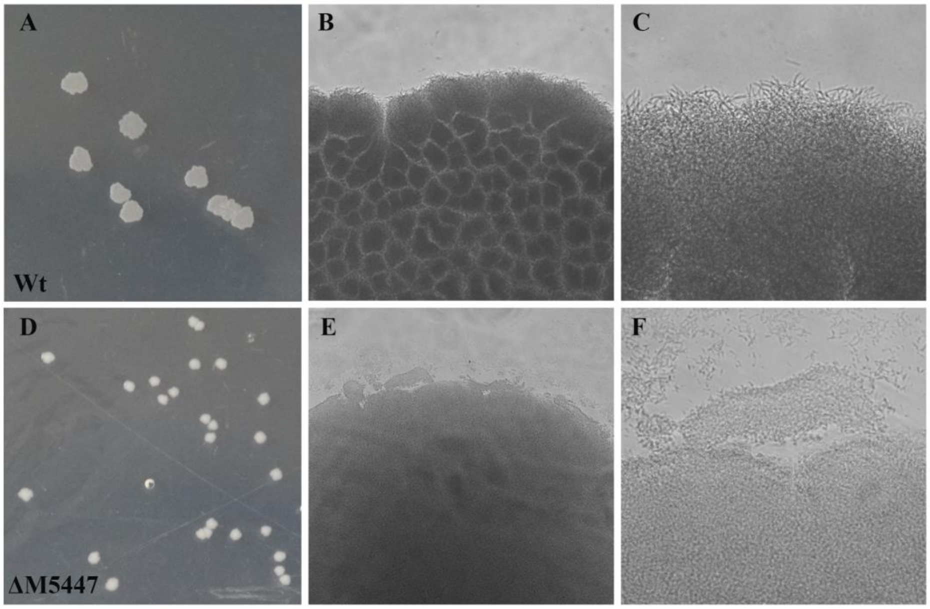

Figure S2. Colony morphology of the Wt and ΔM5447 strains on Middlebrook 7H11 agar plates. The colonies of Wt and ΔM5447 mutant were showed on 7H11 agar plate (A and D). The morphology of single colony was observed by optical microscope at 100× (B and E) and 400×(C and F) respectively.

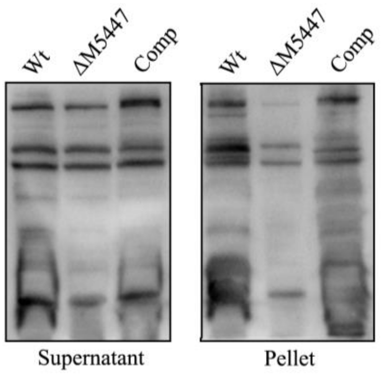

Figure S3. Inactivation of MSMEG_5447 gene interrupts the O-mannosylation of proteins of *M. smegmatis*. *M. smegmatis* mc^{2}155 (Wt), ΔM5447 and Comp strains were grown in LBT broth for 36 h. After the bacterial pellet was lysed by sonication, the bacterial supernatant and pellet were obtained by centrifugation. The level of O-mannosylation of proteins was analyzed by ConA lectin blotting.

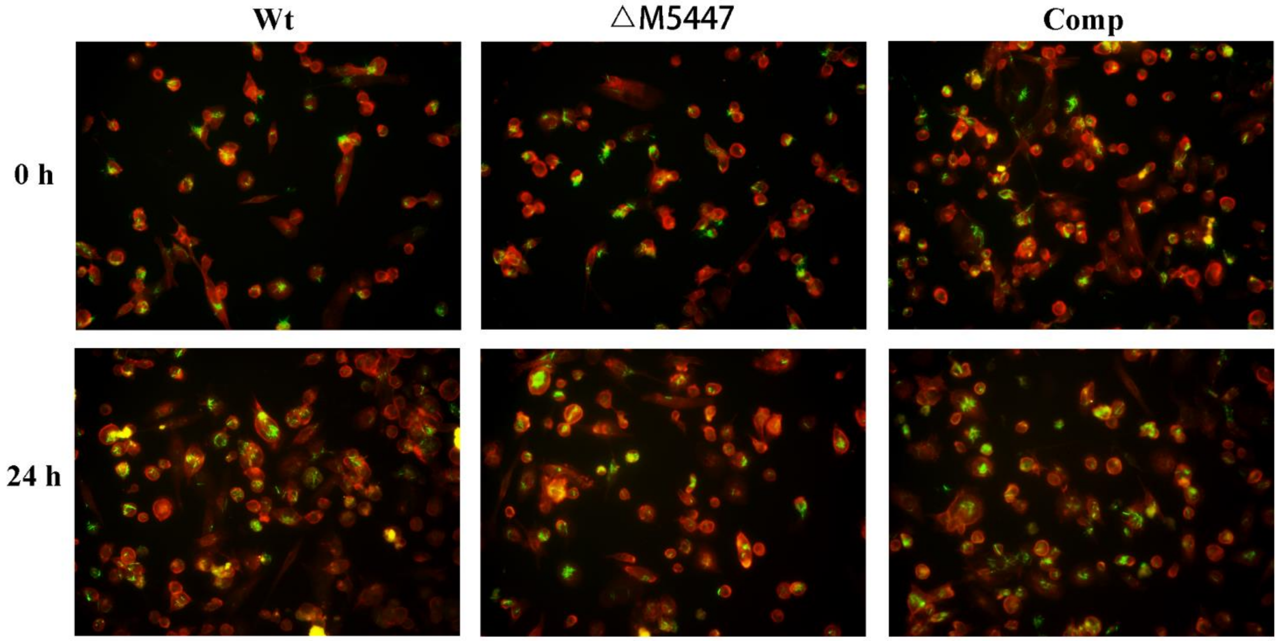

Figure S4. ΔM5447 displayed impairment in survival ability in THP-1 cells. The mycobacterial invasion and survival rate were evaluated through mycobacteria-GFP in cells at 0 h and 24 h of post-infection.

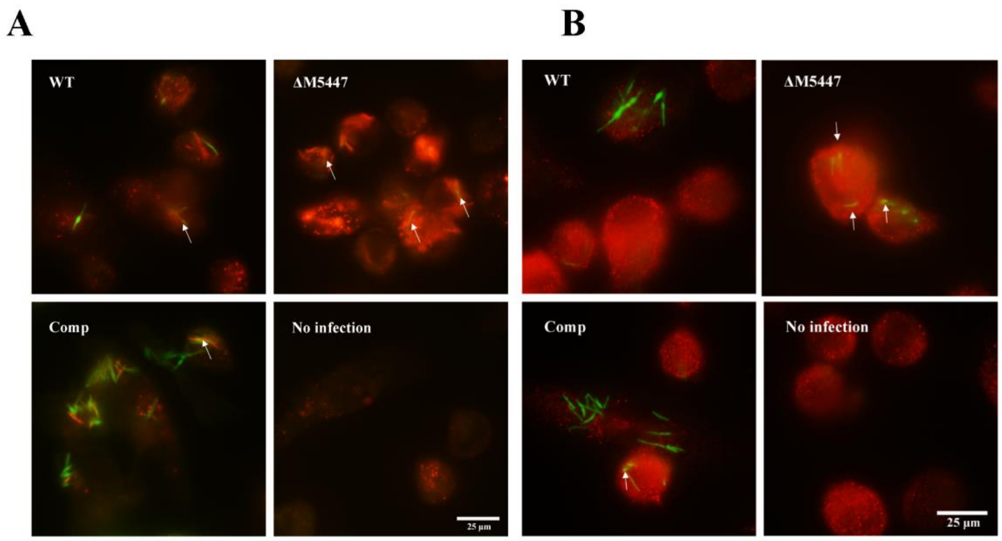

Figure S5. ΔM5447 stain failed to arrest the phagosome-lysosome fusion in infected macrophages. THP-1 macrophages were infected with green fluorescence protein (GFP)-expressing Wt, ΔM5447 and Comp strain for 3h at a MOI of 10. After 24 h infection, cells were stained with LysoTracker Red for 30 min before fixation **(A)** or incubated with LAMP-1 antibody **(B)**. The cells were visualized by fluorescence microscope. The yellow represented the co-localization of green and red fluorescence as shown in images. The white arrows indicate the co-localization of green and red fluorescence. Scale bars, 25 µm

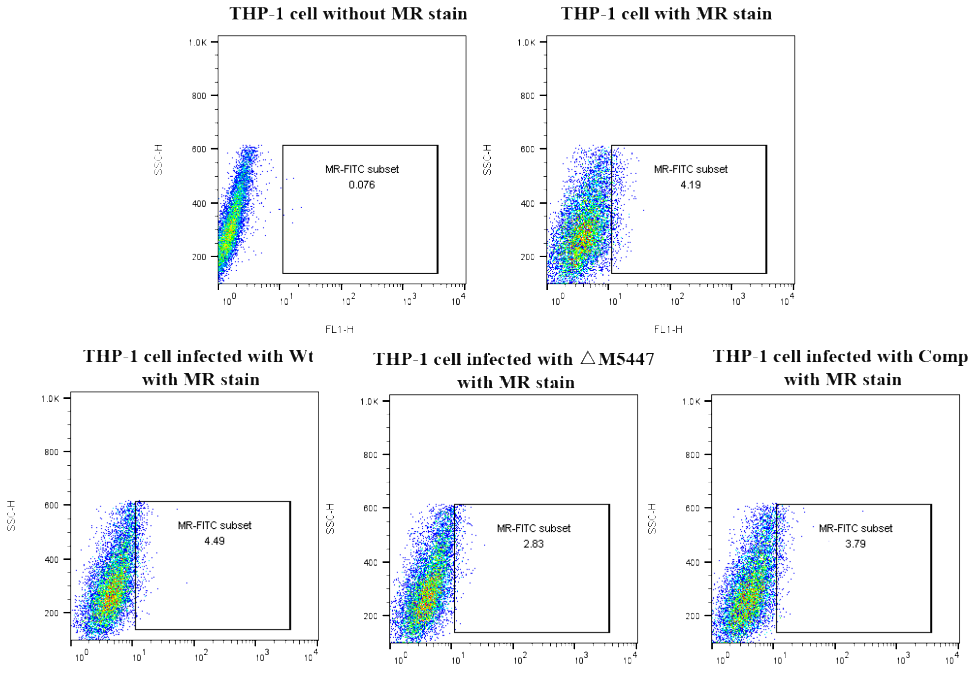

Figure S6. The expression of MR was decreased in ΔM5447-infected macrophages. Macrophage cells were infected with Wt, ΔM5447 and Comp strain at a MOI of 10. The cells were harvest at 0 h of post-infection and subsequently stained with anti-CD206. The expression of MR was analyzed by flow cytometry. The percentage of cells in each square of the flow plot is indicated.

1.2 Table S1. The list of differentially expressed genes (DEGs). THP-1 macrophages were infected with Wt and ΔM5447 for 3 h, respectively. The transcriptomic analysis of macrophages was performed at 24 h post-infection. DEGs were identified by using the Deseq2 package and adjusted $p < 0.05$ was adopted as the criteria. Each group was performed in duplicate.
