# Supplementary material for: Effect of Protein O-Mannosyltransferase (MSMEG_5447) on M. smegmatis and Its Survival in Macrophages
Source: Front Microbiol. 2021 Jun 30;12:657726. doi: 10.3389/fmicb.2021.657726 (PMC8278756; doi:10.3389/fmicb.2021.657726)
Supplement: Supplementary file 2 [file Data_Sheet_2.PDF]

Supplementary Table S1. The list of differentially expressed genes

gene_name	gene_description
EIF3C	eukaryotic translation initiation factor 3 subunit C
AC004057.1	transcribed process
TNF	tumor necrosis factor
APOBEC3A	apolipoprotein B mRNA editing enzyme catalytic subunit 3A
BHLHE40	basic helix-loop-helix family member e40
ZC3H12C	zinc finger CCCH-type containing 12C
NR4A3	nuclear receptor subfamily 4 group A member 3
TNFAIP3	TNF alpha induced protein 3
MSC	musculin
BCL3	B-cell CLL/lymphoma 3
PDE4B	phosphodiesterase 4B
GADD45B	growth arrest and DNA damage inducible beta
NFKBIA	NFKB inhibitor alpha
WTAP	WT1 associated protein
JUNB	JunB proto-oncogene, AP-1 transcription factor subunit
NFKBIZ	NFKB inhibitor zeta
ABL2	ABL proto-oncogene 2, non-receptor tyrosine kinase
OTUD1	OTU deubiquitinase 1
PPP1R15A	protein phosphatase 1 regulatory subunit 15A
CEBPA	CCAAT/enhancer binding protein alpha
GPR132	G protein-coupled receptor 132
LRP1	LDL receptor related protein 1
SDS	serine dehydratase
PARP14	poly(ADP-ribose) polymerase family member 14
DUSP2	dual specificity phosphatase 2
KLF10	Kruppel like factor 10
MFSD2A	major facilitator superfamily domain containing 2A
BTG2	BTG anti-proliferation factor 2
PNRC1	proline rich nuclear receptor coactivator 1
IRAK2	interleukin 1 receptor associated kinase 2
ETS2	ETS proto-oncogene 2, transcription factor
CXCL1	C-X-C motif chemokine ligand 1
IFNB1	interferon beta 1
SOCS3	suppressor of cytokine signaling 3
OGFRL1	opioid growth factor receptor like 1
BTG1	BTG anti-proliferation factor 1
DUSP10	dual specificity phosphatase 10
KLF6	Kruppel like factor 6
IER3	immediate early response 3
CD80	CD80 molecule
SOD2	superoxide dismutase 2
RICTOR	RPTOR independent companion of MTOR complex 2
VEGFA	vascular endothelial growth factor A
SRGN	serglycin
NR4A2	nuclear receptor subfamily 4 group A member 2
PABPC4	poly(A) binding protein cytoplasmic 4
MAFF	MAF bZIP transcription factor F
HIF1A	hypoxia inducible factor 1 alpha subunit
REL	REL proto-oncogene, NF-kB subunit
ST14	suppression of tumorigenicity 14
CXCL3	C-X-C motif chemokine ligand 3
NISCH	nischarin
GEM	GTP binding protein overexpressed in skeletal muscle
CDC42EP2	CDC42 effector protein 2
DOK2	docking protein 2 [
TNFAIP6	TNF alpha induced protein 6

SLFN5	schlafen family member 5
CXCL2	C-X-C motif chemokine ligand 2
ZC3H12A	zinc finger CCCH-type containing 12A
SMIM4	small integral membrane protein 4
ZBTB43	zinc finger and BTB domain containing 43
PHLDA1	pleckstrin homology like domain family A member 1
PLA2G15	phospholipase A2 group XV
CPEB2	cytoplasmic polyadenylation element binding protein 2
CCDC85B	coiled-coil domain containing 85B
FAM78A	family with sequence similarity 78 member A
RASGEF1B	RasGEF domain family member 1B
XBP1	X-box binding protein 1
HIVEP2	human immunodeficiency virus type I enhancer binding protein 2
CCL2	C-C motif chemokine ligand 2
MIDN	midnolin
ZNF697	zinc finger protein 697
PLCB2	phospholipase C beta 2
NFKB1	nuclear factor kappa B subunit 1
CARD9	caspase recruitment domain family member 9
PLAU	plasminogen activator, urokinase
CXCL12	C-X-C motif chemokine ligand 12
RLF	rearranged L-myc fusion
ANKRD1	ankyrin repeat domain 1
NRP2	neuropilin 2
CORO1A	coronin 1A
RGS2	regulator of G protein signaling 2
NEURL3	neuralized E3 ubiquitin protein ligase 3
CFLAR	CASP8 and FADD like apoptosis regulator
GLUL	glutamate-ammonia ligase
BIRC3	baculoviral IAP repeat containing 3
FOXO3	forkhead box O3
ICAM1	intercellular adhesion molecule 1
IER5	immediate early response 5
HBEGF	heparin binding EGF like growth factor
S100A4	S100 calcium binding protein A4
DDIT4	DNA damage inducible transcript 4
CD44	CD44 molecule (Indian blood group)
PLAUR	plasminogen activator, urokinase receptor
SH2B2	SH2B adaptor protein 2
CHD2	chromodomain helicase DNA binding protein 2
PTGER4	prostaglandin E receptor 4
ALOX5	arachidonate 5-lipoxygenase
RND3	Rho family GTPase 3
PIM3	Pim-3 proto-oncogene, serine/threonine kinase
GPX1	glutathione peroxidase 1
ESYT1	extended synaptotagmin 1
APBB1IP	amyloid beta precursor protein binding family B member 1 interacting protein
NFE2L2	nuclear factor, erythroid 2 like 2
CXCL11	C-X-C motif chemokine ligand 11
SLC7A11	solute carrier family 7 member 11
NABP1	nucleic acid binding protein 1
TNIP1	TNFAIP3 interacting protein 1
METRNL	meteorin like, glial cell differentiation regulator
ALOX5AP	arachidonate 5-lipoxygenase activating protein
MEF2D	myocyte enhancer factor 2D
QPRT	quinolinate phosphoribosyltransferase
DHCR24	24-dehydrocholesterol reductase
DMXL2	Dmx like 2

RNF166	ring finger protein 166
ZFP36	ZFP36 ring finger protein
GPR183	G protein-coupled receptor 183
LRRC25	leucine rich repeat containing 25
KLF9	Kruppel like factor 9
CD109	CD109 molecule
SLC2A4RG	SLC2A4 regulator
TWIST1	twist family bHLH transcription factor 1
PMAIP1	phorbol-12-myristate-13-acetate-induced protein 1
TPCN1	two pore segment channel 1
TNRC18	trinucleotide repeat containing 18
SH2B3	SH2B adaptor protein 3
RXRA	retinoid X receptor alpha
VSIR	V-set immunoregulatory receptor
GCH1	GTP cyclohydrolase 1
FNIP2	folliculin interacting protein 2
GAMT	guanidinoacetate N-methyltransferase
CD274	CD274 molecule
KDM6B	lysine demethylase 6B
DGKZ	diacylglycerol kinase zeta
ARID5B	AT-rich interaction domain 5B
ATP13A3	ATPase 13A3
TRAF3IP2	TRAF3 interacting protein 2
IDH2	isocitrate dehydrogenase (NADP(+)) 2, mitochondrial
FAM107B	family with sequence similarity 107 member B
ELL2	elongation factor for RNA polymerase II 2
APEX1	apurinic/apyrimidinic endodeoxyribonuclease 1
MED13	mediator complex subunit 13
CPT1A	carnitine palmitoyltransferase 1A
NCOA4	nuclear receptor coactivator 4
SAT1	spermidine/spermine N1-acetyltransferase 1
SPN	sialophorin
SLC7A8	solute carrier family 7 member 8
MAPK6	mitogen-activated protein kinase 6
ETS1	ETS proto-oncogene 1, transcription factor
IFIT3	interferon induced protein with tetratricopeptide repeats 3
PAPD7	poly(A) RNA polymerase D7, non-canonical
COLGALT1	collagen beta(1-O)galactosyltransferase 1
CD209	CD209 molecule
IFI44L	interferon induced protein 44 like
PDLIM7	PDZ and LIM domain 7
ZSWIM4	zinc finger SWIM-type containing 4
ANXA5	annexin A5
ARL5B	ADP ribosylation factor like GTPase 5B
RRM2B	ribonucleotide reductase regulatory TP53 inducible subunit M2B
MAP2K3	mitogen-activated protein kinase kinase 3
KIF1B	kinesin family member 1B
COTL1	coactosin like F-actin binding protein 1
SLC27A3	solute carrier family 27 member 3
ABTB2	ankyrin repeat and BTB domain containing 2
LMO4	LIM domain only 4
SASH3	SAM and SH3 domain containing 3
CSRNP1	cysteine and serine rich nuclear protein 1
STAT4	signal transducer and activator of transcription 4
ETV3	ETS variant 3
RPS6KA1	ribosomal protein S6 kinase A1
ACSL1	acyl-CoA synthetase long chain family member 1
AZIN1	antizyme inhibitor 1

TLR2	toll like receptor 2
KYNU	kynureninase
PNKD	paroxysmal nonkinesigenic dyskinesia
TREM2	triggering receptor expressed on myeloid cells 2
SLA	Src like adaptor
JMJD1C	jumonji domain containing 1C
SLAMF7	SLAM family member 7
TUBA1A	tubulin alpha 1a
PTPRE	protein tyrosine phosphatase, receptor type E
ZEB2	zinc finger E-box binding homeobox 2
BCL6	B-cell CLL/lymphoma 6
ADGRE5	adhesion G protein-coupled receptor E5
SH3TC1	SH3 domain and tetratricopeptide repeats 1
SDC4	syndecan 4
UBALD2	UBA like domain containing 2
PIK3CD	phosphatidylinositol-4,5-bisphosphate 3-kinase catalytic subunit delta
SKIL	SKI like proto-oncogene
MAMLD1	mastermind like domain containing 1
SAMD9	sterile alpha motif domain containing 9
ASAP1	ArfGAP with SH3 domain, ankyrin repeat and PH domain 1
TTC7A	tetratricopeptide repeat domain 7A
MALT1	MALT1 paracaspase
ITGB8	integrin subunit beta 8
PTPRC	protein tyrosine phosphatase, receptor type C
NFKB2	nuclear factor kappa B subunit 2
ZCCHC2	zinc finger CCHC-type containing 2
USP12	ubiquitin specific peptidase 12
TBC1D13	TBC1 domain family member 13
TMEM123	transmembrane protein 123
PKIG	cAMP-dependent protein kinase inhibitor gamma
TNFAIP2	TNF alpha induced protein 2
PDE4DIP	phosphodiesterase 4D interacting protein
VIM	vimentin
PPIF	peptidylprolyl isomerase F
KITLG	KIT ligand
TNFAIP8	TNF alpha induced protein 8
FRMD4A	FERM domain containing 4A
NAMPT	nicotinamide phosphoribosyltransferase
SLC2A5	solute carrier family 2 member 5
CCNL1	cyclin L1
SRSF5	serine and arginine rich splicing factor 5
SLC38A2	solute carrier family 38 member 2
FAM60A	family with sequence similarity 60 member A
ACSL4	acyl-CoA synthetase long chain family member 4
SMAD7	SMAD family member 7
NOTCH1	notch 1
TP53INP2	tumor protein p53 inducible nuclear protein 2
PPP1R15B	protein phosphatase 1 regulatory subunit 15B
CREBRF	CREB3 regulatory factor
PFKFB3	6-phosphofructo-2-kinase/fructose-2,6-biphosphatase 3
ADPRHL1	ADP-ribosylhydrolase like 1
NAPRT	nicotinate phosphoribosyltransferase
PTP4A1	protein tyrosine phosphatase type IVA, member 1
SLC1A5	solute carrier family 1 member 5
SRM	spermidine synthase
TMC6	transmembrane channel like 6
SMIM3	small integral membrane protein 3
TIPARP	TCDD inducible poly(ADP-ribose) polymerase

RALA	RAS like proto-oncogene A
PELI1	pellino E3 ubiquitin protein ligase 1
RNF187	ring finger protein 187
CASP3	caspase 3
LIMD2	LIM domain containing 2
TUBA1B	tubulin alpha 1b
ARHGAP21	Rho GTPase activating protein 21
TANK	TRAF family member associated NFKB activator
RFTN1	raftlin, lipid raft linker 1
B4GALT1	beta-1,4-galactosyltransferase 1
CXCL10	C-X-C motif chemokine ligand 10
SESN2	sestrin 2
SLC25A6	solute carrier family 25 member 6
SLC1A3	solute carrier family 1 member 3
EIF4EBP1	eukaryotic translation initiation factor 4E binding protein 1
PTPN12	protein tyrosine phosphatase, non-receptor type 12
UBE2Z	ubiquitin conjugating enzyme E2 Z
EIF3G	eukaryotic translation initiation factor 3 subunit G
DDX5	DEAD-box helicase 5
GLIS3	GLIS family zinc finger 3
RUNX1	runt related transcription factor 1
TOR4A	torsin family 4 member A
WASHC4	WASH complex subunit 4
USP15	ubiquitin specific peptidase 15
PARP1	poly(ADP-ribose) polymerase 1
BCOR	BCL6 corepressor
FSCN1	fascin actin-bundling protein 1
KLHL24	kelch like family member 24
KCNAB2	potassium voltage-gated channel subfamily A regulatory beta subunit 2
RDX	radixin
SP3	Sp3 transcription factor
UGCG	UDP-glucose ceramide glucosyltransferase
SNX10	sorting nexin 10
CAPG	capping actin protein, gelsolin like
RPSA	ribosomal protein SA
STAG2	stromal antigen 2
STK17A	serine/threonine kinase 17a
AFF4	AF4/FMR2 family member 4
CLK1	CDC like kinase 1
COL4A2	collagen type IV alpha 2 chain
PFN1	profilin 1
RAP1B	RAP1B, member of RAS oncogene family
PRR12	proline rich 12
RUNX2	runt related transcription factor 2
LYL1	LYL1, basic helix-loop-helix family member
BLVRB	biliverdin reductase B
IRS2	insulin receptor substrate 2
SAMD9L	sterile alpha motif domain containing 9 like
MIF4GD	MIF4G domain containing
PYCARD	PYD and CARD domain containing
C1QA	complement C1q A chain
UXS1	UDP-glucuronate decarboxylase 1
GCLC	glutamate-cysteine ligase catalytic subunit
RSAD2	radical S-adenosyl methionine domain containing 2
RNH1	ribonuclease/angiogenin inhibitor 1
UBA52	ubiquitin A-52 residue ribosomal protein fusion product 1
RPL29	ribosomal protein L29
TCF3	transcription factor 3

IGFBP3	insulin like growth factor binding protein 3
DOT1L	DOT1 like histone lysine methyltransferase
VHL	von Hippel-Lindau tumor suppressor
SOX12	SRY-box 12
TOLLIP	toll interacting protein
MCOLN2	mucolipin 2
FBXO32	F-box protein 32
CD83	CD83 molecule
PRDM2	PR/SET domain 2
SH2D3C	SH2 domain containing 3C
FES	FES proto-oncogene, tyrosine kinase
CAMK1	calcium/calmodulin dependent protein kinase I
NCF1	neutrophil cytosolic factor 1
GRK2	G protein-coupled receptor kinase 2
SCAMP5	secretory carrier membrane protein 5
MAZ	MYC associated zinc finger protein
PIM2	Pim-2 proto-oncogene, serine/threonine kinase
KIAA0930	KIAA0930
IQGAP1	IQ motif containing GTPase activating protein 1
PRDM1	PR/SET domain 1
CTSS	cathepsin S
SLC29A1	solute carrier family 29 member 1
SH3BP5L	SH3 binding domain protein 5 like
GNA13	G protein subunit alpha 13
CTSB	cathepsin B
C7orf50	chromosome 7 open reading frame 50
HES4	hes family bHLH transcription factor 4
AARS	alanyl-tRNA synthetase
DOK3	docking protein 3
B2M	beta-2-microglobulin
RPL37A	ribosomal protein L37a
NT5DC2	5'-nucleotidase domain containing 2
AHR	aryl hydrocarbon receptor
GSAP	gamma-secretase activating protein
HERC6	HECT and RLD domain containing E3 ubiquitin protein ligase family member 6
TBX3	T-box 3
DDX60L	DEAD-box helicase 60 like
RGS16	regulator of G protein signaling 16
ABCA2	ATP binding cassette subfamily A member 2
SSH1	slingshot protein phosphatase 1
RPS29	ribosomal protein S29
XRN1	5'-3' exoribonuclease 1
MASTL	microtubule associated serine/threonine kinase like
EGR2	early growth response 2
SIK3	SIK family kinase 3
GBP1	guanylate binding protein 1
HK2	hexokinase 2
DDX58	DExD/H-box helicase 58
GRAMD4	GRAM domain containing 4
RASSF8	Ras association domain family member 8
JUN	Jun proto-oncogene, AP-1 transcription factor subunit
NBN	nibrin
GBP5	guanylate binding protein 5
PRDX2	peroxiredoxin 2
PITPNA	phosphatidylinositol transfer protein alpha
PERP	PERP, TP53 apoptosis effector
HCN2	hyperpolarization activated cyclic nucleotide gated potassium and sodium channel 2
NAPSB	napsin B aspartic peptidase, pseudogene

GRAMD1A	GRAM domain containing 1A
PARM1	prostate androgen-regulated mucin-like protein 1
IL1R1	interleukin 1 receptor type 1
TRPM2	transient receptor potential cation channel subfamily M member 2
IL17RA	interleukin 17 receptor A
FTH1	ferritin heavy chain 1
KPNB1	karyopherin subunit beta 1
FLOT2	flotillin 2
AHCY	adenosylhomocysteinase
BCL2	BCL2, apoptosis regulator
CLUH	clustered mitochondria homolog
MIR3142HG	MIR3142 host gene
NIPBL	NIPBL, cohesin loading factor
DDX3X	DEAD-box helicase 3, X-linked
INPP5D	inositol polyphosphate-5-phosphatase D
ARRDC3	arrestin domain containing 3
RAPGEF2	Rap guanine nucleotide exchange factor 2
DDT	D-dopachrome tautomerase
TWF2	twinfilin actin binding protein 2
SCAP	SREBF chaperone
SPAG9	sperm associated antigen 9
RPLP0	ribosomal protein lateral stalk subunit P0
ARID5A	AT-rich interaction domain 5A
MAP4K4	mitogen-activated protein kinase kinase kinase kinase 4
HPCAL1	hippocalcin like 1
SH3GL1	SH3 domain containing GRB2 like 1, endophilin A2
MS4A7	membrane spanning 4-domains A7
OLFML3	olfactomedin like 3
SCIMP	SLP adaptor and CSK interacting membrane protein
CCL4L2	C-C motif chemokine ligand 4 like 2
MGLL	monoglyceride lipase
RAPGEF1	Rap guanine nucleotide exchange factor 1
CYFIP1	cytoplasmic FMR1 interacting protein 1
ALCAM	activated leukocyte cell adhesion molecule
LIMS1	LIM zinc finger domain containing 1
EIF5	eukaryotic translation initiation factor 5
RPL18A	ribosomal protein L18a
MCL1	MCL1, BCL2 family apoptosis regulator
DUSP23	dual specificity phosphatase 23
ADAM9	ADAM metallopeptidase domain 9
UBE2D3	ubiquitin conjugating enzyme E2 D3
NCOA7	nuclear receptor coactivator 7
TXNRD1	thioredoxin reductase 1
SIDT2	SID1 transmembrane family member 2
FASN	fatty acid synthase
BLZF1	basic leucine zipper nuclear factor 1
AL390728.4	
NINJ1	ninjurin 1
MMS19	MMS19 homolog, cytosolic iron-sulfur assembly component
SOWAHD	sosondowah ankyrin repeat domain family member D
CTNNB1	catenin beta 1
PARP4	poly(ADP-ribose) polymerase family member 4
CXCL8	C-X-C motif chemokine ligand 8
ZBTB10	zinc finger and BTB domain containing 10
RRP7A	ribosomal RNA processing 7 homolog A
TET2	tet methylcytosine dioxygenase 2
ATP5G3	ATP synthase, H+ transporting, mitochondrial Fo complex subunit C3 (subunit 9)
LYN	LYN proto-oncogene, Src family tyrosine kinase

ZFP91	ZFP91 zinc finger protein
TSPO	translocator protein
AC006449.6	
TFEC	transcription factor EC
FAM20C	FAM20C, golgi associated secretory pathway kinase
SGK1	serum/glucocorticoid regulated kinase 1
ABHD8	abhydrolase domain containing 8
CLIC4	chloride intracellular channel 4
SERTAD2	SERTA domain containing 2
AGO2	argonaute 2, RISC catalytic component
CMTM3	CKLF like MARVEL transmembrane domain containing 3
SNX9	sorting nexin 9
IFRD1	interferon related developmental regulator 1
ITGAV	integrin subunit alpha V
CRTAP	cartilage associated protein
LCP2	lymphocyte cytosolic protein 2
CCL4	C-C motif chemokine ligand 4
H1FX	H1 histone family member X
NFAT5	nuclear factor of activated T-cells 5
PSD3	pleckstrin and Sec7 domain containing 3
TNFAIP8L2	TNF alpha induced protein 8 like 2
STX11	syntaxin 11
AL353625.1	
PTMS	parathymosin
ANKRD13D	ankyrin repeat domain 13D
RPL8	ribosomal protein L8
CD40	CD40 molecule
C5AR1	complement C5a receptor 1
TECR	trans-2,3-enoyl-CoA reductase
ST8SIA4	ST8 alpha-N-acetyl-neuraminide alpha-2,8-sialyltransferase 4
ASS1	argininosuccinate synthase 1
C2CD2L	C2CD2 like
IFIT2	interferon induced protein with tetratricopeptide repeats 2
LIMK1	LIM domain kinase 1
TKT	transketolase
ZNRF2	zinc and ring finger 2
PARVG	parvin gamma
ARHGAP4	Rho GTPase activating protein 4
RNPEPL1	arginyl aminopeptidase like 1
YWHAQ	tyrosine 3-monooxygenase/tryptophan 5-monooxygenase activation protein theta
ANKRD33B	ankyrin repeat domain 33B
SDCBP	syndecan binding protein
GBP3	guanylate binding protein 3
PRLR	prolactin receptor
IFIH1	interferon induced with helicase C domain 1
MID1IP1	MID1 interacting protein 1
FARSA	phenylalanyl-tRNA synthetase alpha subunit
PDLIM2	PDZ and LIM domain 2
PDLIM1	PDZ and LIM domain 1
RPP25	ribonuclease P and MRP subunit p25
IRGQ	immunity related GTPase Q
ARHGEF3	Rho guanine nucleotide exchange factor 3
RPS2	ribosomal protein S2
RPL37	ribosomal protein L37
LSS	lanosterol synthase
SPNS2	sphingolipid transporter 2
P2RY6	pyrimidinergic receptor P2Y6
TEAD1	TEA domain transcription factor 1

LIFR	LIF receptor alpha
MAF	MAF bZIP transcription factor
RPL13	ribosomal protein L13
TSPAN14	tetraspanin 14
MYL9	myosin light chain 9
ABI1	abl interactor 1
SERINC1	serine incorporator 1
DNASE2	deoxyribonuclease 2, lysosomal
FNDC3B	fibronectin type III domain containing 3B
CAMK2G	calcium/calmodulin dependent protein kinase II gamma
ANKRD10	ankyrin repeat domain 10
NAXE	NAD(P)HX epimerase
PITX1	paired like homeodomain 1
TAB2	TGF-beta activated kinase 1/MAP3K7 binding protein 2
PMVK	phosphomevalonate kinase
LCP1	lymphocyte cytosolic protein 1
NECTIN3	nectin cell adhesion molecule 3
NLRP3	NLR family pyrin domain containing 3
SCAMP2	secretory carrier membrane protein 2
ZYX	zyxin
PNRC2	proline rich nuclear receptor coactivator 2
DUSP8	dual specificity phosphatase 8
ATP8B4	ATPase phospholipid transporting 8B4 (putative)
TBK1	TANK binding kinase 1
PLEK	pleckstrin
ISOC2	isochorismatase domain containing 2
SLC12A9	solute carrier family 12 member 9
TMEM104	transmembrane protein 104
LPCAT1	lysophosphatidylcholine acyltransferase 1
RASSF5	Ras association domain family member 5
PYGO2	pygopus family PHD finger 2
ARMC9	armadillo repeat containing 9
PLAC8	placenta specific 8
CD55	CD55 molecule (Cromer blood group)
PKD1	polycystin 1, transient receptor potential channel interacting
